# *LMPA* Regulates Lesion Mimic Leaf and Panicle Development Through ROS-Induced PCD in Rice

**DOI:** 10.3389/fpls.2022.875038

**Published:** 2022-05-02

**Authors:** Peng Hu, Yiqing Tan, Yi Wen, Yunxia Fang, Yueying Wang, Hao Wu, Junge Wang, Kaixiong Wu, Bingze Chai, Li Zhu, Guangheng Zhang, Zhenyu Gao, Deyong Ren, Dali Zeng, Lan Shen, Dawei Xue, Qian Qian, Jiang Hu

**Affiliations:** ^1^Agricultural Genomics Institute at Shenzhen, Chinese Academy of Agricultural Sciences, Shenzhen, China; ^2^State Key Laboratory of Rice Biology, China National Rice Research Institute, Hangzhou, China; ^3^Rice Research Institute of Shenyang Agricultural University/Key Laboratory of Northern Japonica Rice Genetics and Breeding, Ministry of Education and Liaoning Province, Shenyang, China; ^4^College of Life and Environmental Sciences, Hangzhou Normal University, Hangzhou, China

**Keywords:** rice, panicle apical abortion, lesion mimic, reactive oxygen species, cell death, plasma membrane H^+^-ATPase

## Abstract

Leaf and panicle are important nutrient and yield organs in rice, respectively. Although several genes controlling lesion mimic leaf and panicle abortion have been identified, a few studies have reported the involvement of a single gene in the production of both the traits. In this study, we characterized a panicle abortion mutant, *lesion mimic leaf and panicle apical abortion* (*lmpa*), which exhibits lesions on the leaf and causes degeneration of apical spikelets. Molecular cloning revealed that *LMPA* encodes a proton pump ATPase protein that is localized in the plasma membrane and is highly expressed in leaves and panicles. The analysis of promoter activity showed that the insertion of a fragment in the promoter of *lmpa* caused a decrease in the transcription level. Cellular and histochemistry analysis indicated that the ROS accumulated and cell death occurred in lmpa. Moreover, physiological experiments revealed that *lmpa* was more sensitive to high temperatures and salt stress conditions. These results provide a better understanding of the role of *LMPA* in panicle development and lesion mimic formation by regulating ROS homeostasis.

## Introduction

Rice (*Oryza sativa*) yield is closely related to panicle architecture, including panicle length and grain number (Sakamoto and Matsuoka, 2008; Xing and Zhang, 2010; Huang et al., 2021). During panicle growth and development, abortion at the apex or base of the panicle usually causes the degeneration of floral organs, eventually leading to a severe reduction in grain yield (Heng et al., 2018; Ali et al., 2019).

It is known that nutrient deficiency or adverse environmental conditions may lead to panicle abortion, particularly in the sterile lines of hybrid rice (Tan et al., 2011; Kobayasi et al., 2015; Zhang et al., 2017; Wang Z. et al., 2018). In recent years, several genes and quantitative trait loci (QTL) involved in the regulation of panicle abortion have been identified and characterized in rice (Cheng et al., 2011; Tan et al., 2011; Bai et al., 2015; Heng et al., 2018; Wang et al., 2021; Ali et al., 2022). *TUT1/ES1* encodes a functional SCAR/WAVE protein that is involved in actin polymerization and panicle development. The loss of function leads to a pleiotropic phenotype, including panicle apical degeneration and early leaf senescence (Bai et al., 2015; Rao et al., 2015). *Short panicle 1* (*sp1*) is characterized by a delayed or completely arrested basal panicle, which is generated from the mutation of the putative PTR family transporter that is involved in the regulation of nitrate transport (Li et al., 2009). Transcriptional co-repressor *ASP1* regulates the axillary meristem determinacy and auxin signaling, and its mutation leads to spikelet abortion at the basal portions of the panicle (Yoshida et al., 2012). The mutant *panicle apical abortion1-1* (*paab1-1*) causes the degeneration of the apical portion of panicles, which is caused by the mutations in aluminum-activated malate transporter *OsALMT7* involved in malate transport (Heng et al., 2018). *OsASA* encodes a boric acid channel protein and plays a role in maintaining boron distribution, and the *asa* mutant showed decreased pollen fertility and apical spikelet abortion phenotypes than those exhibited by the wild type (Zhou et al., 2021).

Reactive oxygen species (ROS) are considered to play a dual role in plant biology. It acts as an important signal molecule involved in plant growth and development and is also considered as a toxic by-product of aerobic metabolism (Mhamdi and Van Breusegem, 2018; Waszczak et al., 2018). Disrupting the balance between ROS production and the scavenging cycle results in oxidative bursts that initiate cell death signals and lead to programmed cell death (PCD) (Choudhary et al., 2020). *SPL6* is a transcriptional repressor of the ER stress sensor *IRE1*, which negatively modulates the amplitude and duration of the IRE1-mediated ER stress signaling outputs. The *spl6* causes panicle apical abortion due to the hyperactivation of the endoplasmic reticulum stress sensor *IRE1* and results in cell death (Wang Q. L. et al., 2018). The loss of function of *OsALMT7* results in malate deficiency, which leads to the accumulation of ROS and triggers PCD in the apical spikelets (Heng et al., 2018). *DPS1* encodes a cystathionine β-synthase domain protein, which interacts with the mitochondrial thioredoxin Trx1 and Trx20 and participates in ROS scavenging (Zafar et al., 2019). *OsCIPK31* regulates panicle development by responding to various stresses and phytohormones, and its mutation causes excessive accumulation of ROS and ultimately leads to PCD in rice panicles (Peng et al., 2018). Although the participation of these genes in the regulation of panicle degeneration has been reported previously, the underlying genetic and molecular mechanisms are still poorly understood.

Plasma membrane H^+^-ATPases (PMAs) play an important role in plant growth and development, provide resistance to biotic or abiotic stress factors and other processes, and are one of the critical enzymes crucial to maintaining the plant life process (Falhof et al., 2016). PMAs can pump out protons from the cytosol into the extracellular space and mediate the transport of water, ions, and nutrients across the plasma membranes by generating proton gradients and negative membrane potentials (Lee et al., 2021; Zhang et al., 2021). Ten PMA subtypes (*OsA1*–*OsA10*) have been identified in rice, and they are mainly involved in nutrient uptake (Chang et al., 2009; Loss Sperandio et al., 2011, 2020; Toda et al., 2016). Among them, overexpression of *OsA1* in rice plants promoted nitrogen absorption and assimilation, enhanced the photosynthetic rate, and increased the grain yield significantly (Loss Sperandio et al., 2011; Zhang et al., 2021). *OsA2* is highly responsive to nitrate (NO${}_{3}^{-}$) ions and plays an important role in nitrogen absorption, plant growth, and grain production (Loss Sperandio et al., 2020). *OsA5* and *OsA7* were also induced by NO${}_{3}^{-}$ ions and may be involved in the net flux of NO${}_{3}^{-}$ ions and plant development (Loss Sperandio et al., 2020).

In this study, we reported and characterized a rice mutant with lesion mimic leaf and panicle apical degeneration phenotypes (*lmpa*). Molecular and genetic analyses revealed that *LMPA* encodes a plasma membrane proton pump ATPase protein and plays a vital role in rice growth and development. Our research further revealed that the accumulation of reactive oxygen species (ROS) and triggering of programmed cell death (PCD) lead to panicle apical degeneration and lesion mimic leaf formation, and this finding will provide insights into the molecular biological functions of *LMPA* in panicle and leaf development.

## Materials and Methods

### Plant Materials and Growth Conditions

The rice *lmpa* mutant was obtained from an ethyl methane sulfonate (EMS)-treated population of *japonica* rice (*Oryza sativa*) variety, Zhonghua11 (ZH11). The *lmpa* mutant was back-crossed to ZH11 to produce M_2_ plants in an isogenic background. In this study, ZH11 was used as the wild type (WT) in all the analyses. All rice plants were grown in the field under natural growth conditions in Hangzhou, Zhejiang Province, China. For the temperature treatment experiments, WT and *lmpa* seeds were grown in a chamber (12 h dark/12 h light cycle at 60% humidity) at 20 and 30°C from germination to 2 weeks. For the salt stress tolerance treatments, the germinated seeds of WT and *lmpa* were sown in a 96-well plate. Then the 7-day-old seedlings were grown in Yoshida's culture solution and treated with different concentrations of NaCl for 7 days. Then, the survival rate of the seedlings was determined after 7 days of recovery under normal conditions.

### Paraffin Sectioning

Plant materials were collected and fixed in 70% FAA (50% ethanol, 5% glacial acetic acid, and 5% formaldehyde) overnight. After fixing, the samples were dehydrated in a graded alcohol series, immersed in xylene, embedded in paraffin, and finally sliced into 8-μm thick sections using a rotary microtome (Leica, HistoCore AUTOCUT). After being dewaxed with xylene and rehydrated with decreasing ethanol concentrations, the prepared slices were stained with 1% safranin and 1% fast Green and observed under a microscope (Nikon, ECLIPSE 90i).

### Transmission Electron Microscopy

For TEM, the flag leaves of WT and *lmpa* mutant plants with the lesion phenotype were harvested at tillering stage and fixed in fixation buffer (2.5% glutaraldehyde in 100 mM phosphate buffer, pH 7.4). After fixing, the samples were washed three times with PBS and fixed with 1% OsO_4_ for 1 h, then dehydrated with a graded alcohol series, and embedded with Suprr Kit (Sigma-Aldrich). The specimens were sliced into 70-nm thick sections with ultratome (Leica, EM UC7), stained with uranyl acetate and alkaline lead citrate, and finally observed under a Hitachi-7500 TEM (Tokyo, Japan).

### Histochemical Assay and Physiological Measurements

The flag leaves of WT and *lmpa* mutant plants with the lesion phenotype were used for physiological measurements. The content of malonaldehyde (MDA) and hydrogen peroxide (H_2_O_2_) and the activities of superoxide dismutase (SOD), peroxidase (POD), catalase (CAT), and ascorbate peroxidase (APX) were measured using an assay kit (Suzhou Keming Biotechnology Co, Ltd.) according to the manufacturer's instructions.

Nitroblue tetrazolium (NBT) and 3, 3′-diaminobenzidine staining (DAB) were performed to detect the accumulation of superoxide anion (O${}_{2}^{-}$) and H_2_O_2_, respectively. In brief, fresh leaves were collected and incubated in 0.05% (w/v) NBT or 0.1% (w/v) DAB (pH 5.8) staining buffer overnight at 28°C, decolored with 75% ethanol until all the chlorophyll had been removed, and finally photographed.

For the GUS staining assay, different tissues of transgenic plants were collected and stained in GUS staining buffer for 12–16 h at 37°C. The samples were then cleared in 75% ethanol and photographed. The GUS activity in the root was analyzed by using the GUS gene quantitative detection kit (Coolaber, China, SL7161).

### TUNEL Assay

The flag leaves and apical spikelets of the WT and *lmpa* plants were collected and fixed in the FAA solution overnight. Briefly, the samples were embedded and sliced according to the method described in the section “Paraffin Sectioning.” After rehydrating with ethanol and being treated with proteinase K, the TUNEL assay was performed with a TUNEL Kit (Promega, G3250) according to the manufacturer's instructions. The nuclei were stained with DAPI, and the apoptotic cells were stained with fluorescein-12-dUTP. The blue fluorescence signal of DAPI and green fluorescence signal of fluorescein (TUNEL signal) were observed at 460 and 520 nm, respectively, under a confocal laser scanning microscope (Zeiss, LSM700).

### Map-Based Cloning of *LMPA*

For genetic analysis, the *lmpa* mutant was crossed with the *indica* variety Nanjing 6 (NJ6) to generate the F_1_ plants, and the plants displaying the panicle abortion phenotype were selected as the mapping population from the F_2_ mapping population. For fine gene mapping, new InDel molecular markers were developed by Primer Premier 5 software. *LMPA* was mapped to a 122.6-kb region on chromosome 4, and the genes predicted to be present within this region were amplified and sequenced, and compared between *lmpa* and WT plants. The primers used are listed in Supplementary Table 1.

### RNA Extraction and qRT-PCR Analysis

Total RNA was isolated using AxyPrep™ total RNA Miniprep Kit (Axygen, AP-MN-MS-RNA) from different tissues. The first strand of complementary cDNA was synthesized with ReverTra Ace kit (Toyobo, FSK-101) from 1 μg of total RNA. The cDNA was diluted for qRT-PCR using the SYBR Green PCR Master Mix kit (Applied Biosystems, 4367659) in ABI7900 (Applied Biosystems). The *OsActin* (*Os04g0177600*) gene of rice was used as an internal reference to normalize the gene expression data. Three replicates were performed for all experiments. The cycle threshold (Ct) method was used to calculate the relative amounts of mRNA. The student's *t-*test was used to analyze the significance of the differences. The primers used are listed in Supplementary Table 1.

### Plasmid Construction and Plant Transformation

To create *lmpa* CRISPR lines, the gene-editing constructs were generated using CRISPR/Cas9 technology. The sgRNA targeting the 12th exon of *LMPA* was cloned into the pYLCRISPR-Cas9Pubi-H vector according to the method previously described (Xie et al., 2017). To compare the promoter activity, the 2,300 and 2,733-bp promoter fragments were amplified from WT and *lmpa*, respectively, and cloned into the pCAMBIA1305.1 vector to generate the plasmid *pLMPA*::GUS and *plmpa*::GUS. To generate the overexpression constructs, the coding sequence of *LMPA* was amplified and cloned into the *pUbi*-GFP-*NosT* vector to create the plasmid *pubi::LMPA-GFP*. All the vectors were introduced into the rice callus via *Agrobacterium* (EHA105)-mediated transformation, as previously described (Toki et al., 2006). The sequences of the PCR primers used for vector construction are listed in Supplementary Table 1.

### Subcellular Localization of LMPA

To determine the subcellular localization of LMPA, the *pUbi::LMPA-GFP* vector was transformed into *N. benthamiana* leaves by initiating an *Agrobacterium* infection. The roots of 3-day-old seedlings of *pUbi::LMPA-GFP* transgenic plants were used to observe the subcellular localization of LMPA. GFP fluorescence was detected with LSM 700 confocal microscope (Zeiss). Plasma membranes were stained with NerveRedTM C2 (Coolaber, China, FM4-64).

### Relative Promoter Activity Assays

To determine whether the promoter mutation affects the *LMPA* expression, the promoter fragments of WT and *lmpa* were amplified and cloned into pGreenII 0800-LUC vector. The *LMPA-LUC* and *lmpa-LUC* plasmids were transformed into rice protoplasts, respectively, according to a previously described method (Ruan et al., 2020). Firefly luciferase (fLUC) and Renilla luciferase (rLUC) were measured using the Dual-Luciferase Reporter Assay System (Promega, E1910). The relative luciferase activity was calculated as the ratio of fLUC/rLUC. The primers used are listed in Supplementary Table 1.

## Results

### The *lmpa* Exhibits a Lesion Mimic Leaf and Panicle Apical Abortion Phenotype

**Figure 1:**
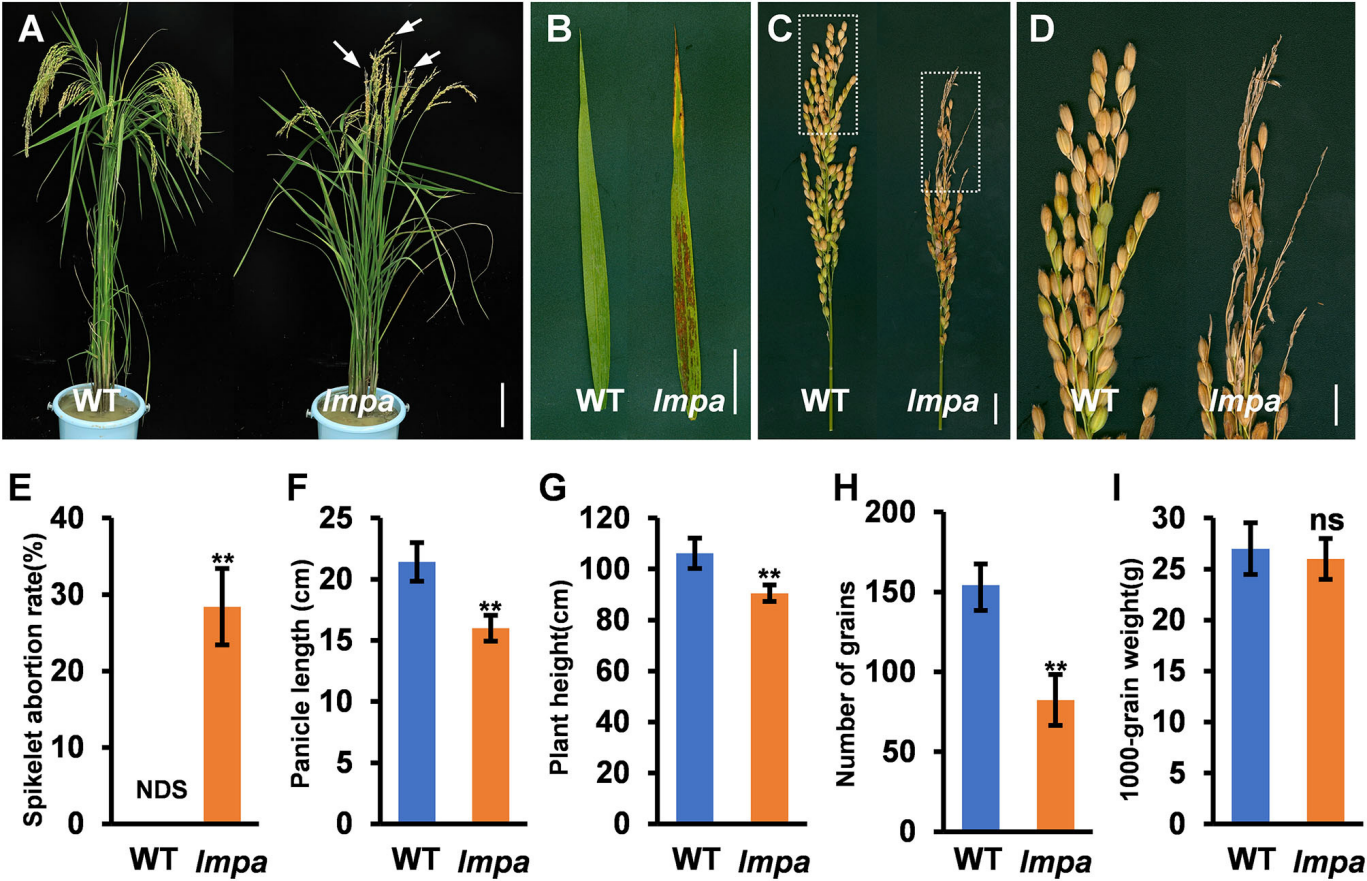
Phenotypic characterization of the *lmpa* mutant. **(A)** Phenotypes of the mature wild-type (WT) plant and *lmpa* mutant. The arrow indicates the degenerated spikelets. **(B)** Flag leaf phenotype in WT and *lmpa* mutant. **(C)** Phenotypic comparison of mature panicles between WT and *lmpa* mutant. **(D)** Magnified view of the white boxed areas in **(C)**. **(E–I)** Comparison of spikelet abortion rate **(E)**, panicle length **(F)**, plant height **(G)**, number of grains per panicle **(H)**, and 1,000-grain weight **(I)** between the WT and *lmpa* mutant. Error bars represent standard deviation (SD) (*n* = 10); **Significant difference at *p* < 0.01 compared with the WT by Student's *t-*test. NDS, no degenerated spikelet. Bars =10 cm in **(A)**, 5 cm in **(B)**, 2 cm in **(C)**, and 1 cm in **(D)**.

The *lmpa* mutant was identified from the EMS mutagenesis library of ZH11. At the seedling stage, the mutant first exhibited white patches on the leaves. Subsequently, lesions appeared and extended to the whole leaf blade with the growth of the plant (Figures 1A,B; Supplementary Figures 1A–D). Moreover, *lmpa* showed severe panicle degeneration at the apical portion of each panicle, and the development of spikelets in the apical part of the panicle stopped and became shriveled (Figures 1A,C–F). The panicle undergoes development in different stages, and the results showed that the whitish spikelets appeared after the panicle reaches a length of 5 cm (Supplementary Figures 2A–F). In addition, the iodine-potassium iodide (I_2_-KI) staining assay displayed that no viable pollen grains were found in the apical spikelets, but the pollen grains of middle and basal spikelets were found to be normal (Supplementary Figures 3A–E). Due to the apical panicle abortion, the panicle length, the number of grains per panicle, and plant height were significantly reduced in lmpa mutants (Figures 1F–H). However, the 1000-grain weight of WT and lmpa has no significant difference (Figure 1I).

### Destruction of Cell Structure in *lmpa*

**Figure 2:**
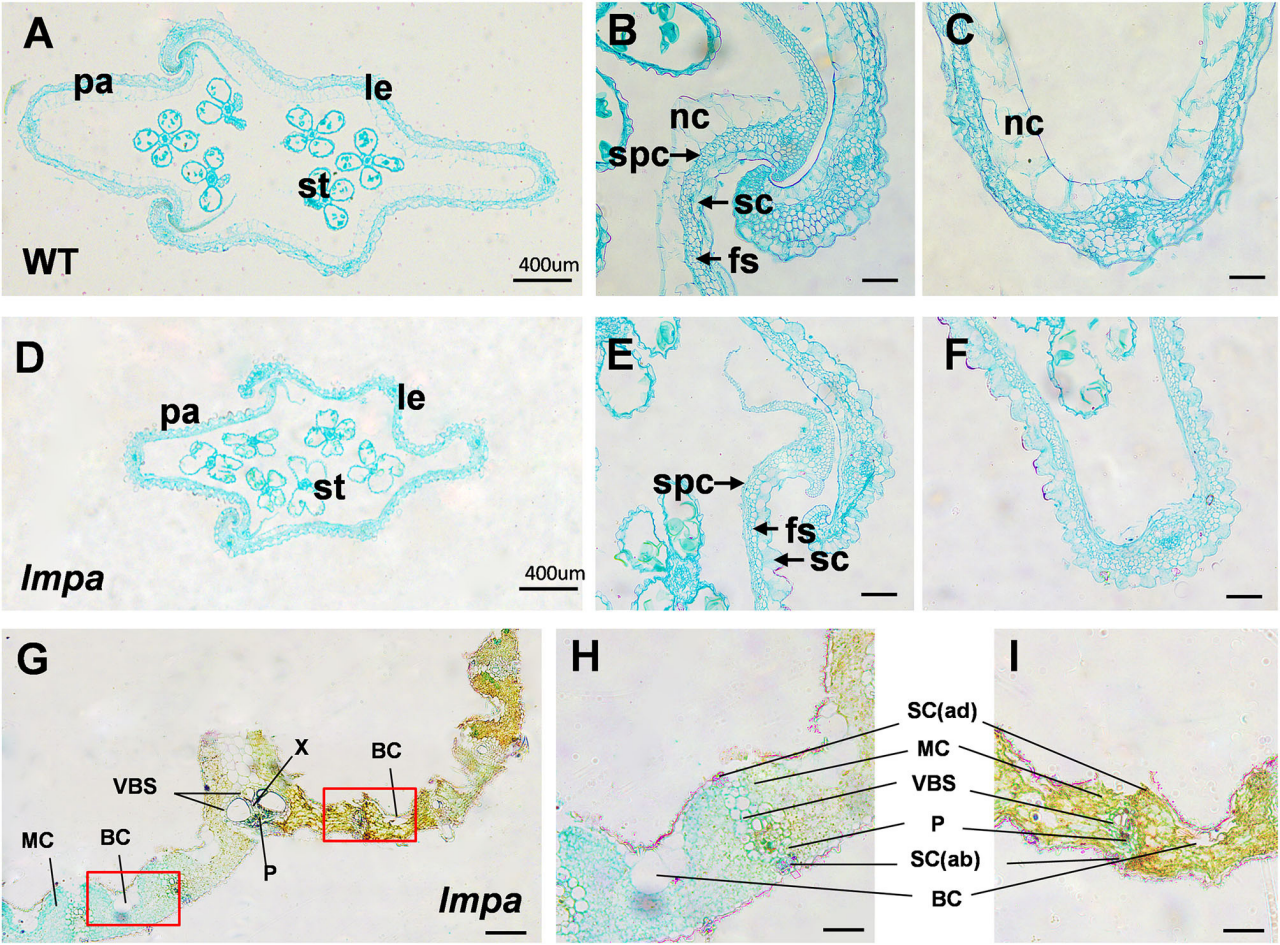
Histological characterization of WT and *lmpa* mutant. **(A–C)** Cross-sections of WT apical spikelet hulls. **(D–F)** Cross-sections of *lmpa* apical spikelet hulls. **(G)** Cross-sections of *lmpa* leaves. **(H)** Normal part of *lmpa* leaves, magnified view of the white boxed areas in **(G)**. **(I)** Spotted part of *lmpa* leaves, magnified view of the white boxed areas in **(G)**. le, lemma; pa, palea; st, stamen; sc, silicified cells; nc, non-silicified cells; fs, fibrous sclerenchyma; spc, spongy parenchymatous cells. SC(ab), abaxial sclerenchyma; BC, bulliform cells; P, phloem; X, xylem; VBS, vascular bundle sheath; MC, mesophyll cells; SC(ad), adaxial sclerenchyma. Bars= 400 μm in **(A,D,G)**, 50 μm in **(B,C,E,F,H,I)**.

To reveal the abnormalities associated with lesion leaf and apical spikelets, a detailed cell structure was investigated by observing the paraffin sections. The results showed that the non-silicified cells were absent in *lmpa* glume (Figures 2A–F). Moreover, the morphological structures between the region of the lesion and normal regions also showed significant differences, including distorted bulliform cells, vascular bundle sheath cells, and mesophyll cells in lesion parts (Figures 2G–I). TEM analysis showed that the matrix thylakoids and stroma lamellae structures of chloroplast were disorderly arranged, and more osmiophilic granules were found in *lmpa*. In addition, the content of Chlorophyll a (chl a), Chlorophyll b (chl b), and carotenoids (car) were remarkably decreased in *lmpa* compared to that of WT (Supplementary Figures 4A–E).

### Cell Death and ROS Accumulation in *lmpa*

**Figure 3:**
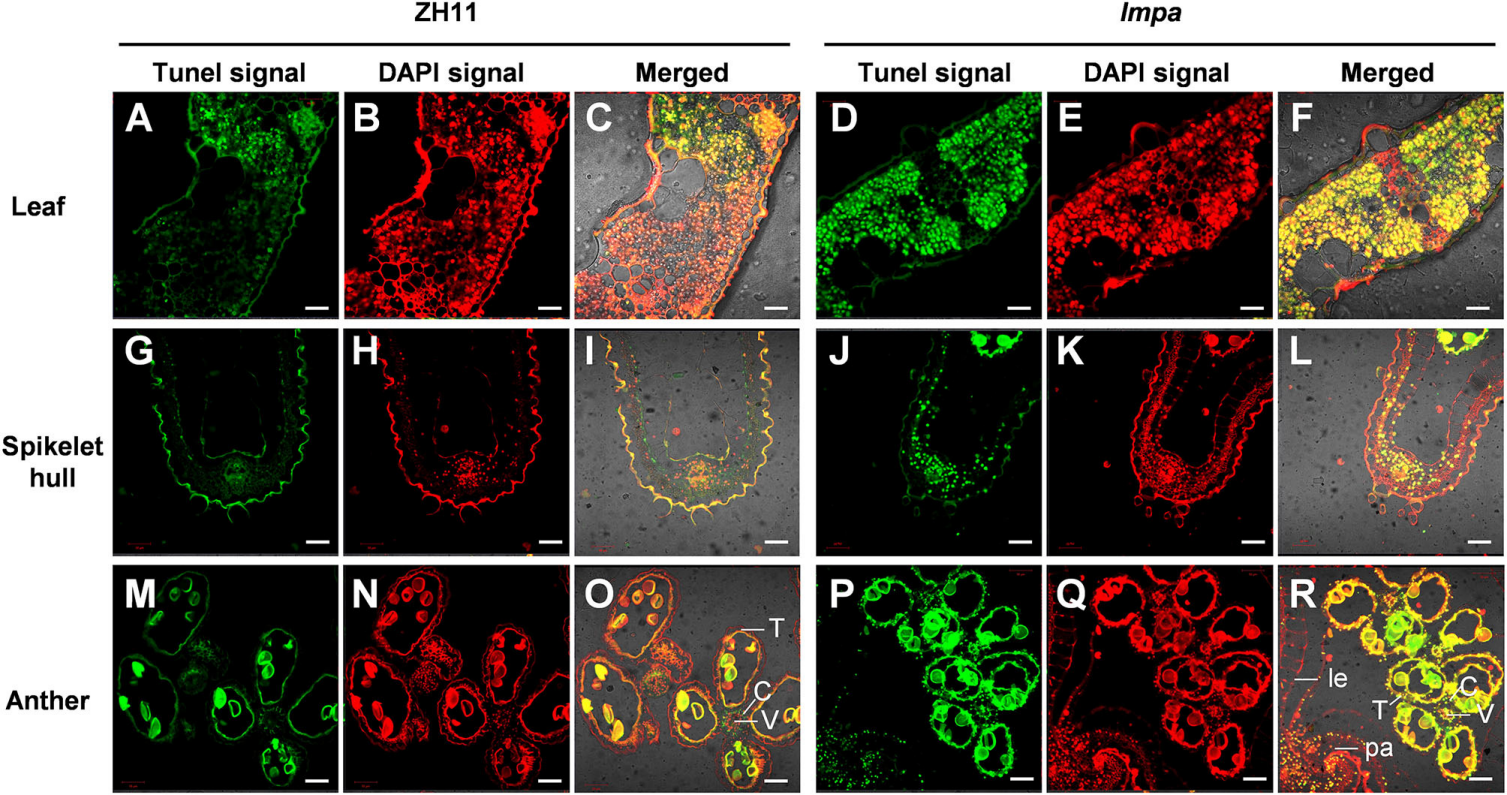
Detection of DNA fragmentation by the TUNEL assay. **(A–F)** TUNEL assay of leaves in WT **(A–C)** and *lmpa* mutant **(D–F)**. **(G–L)** TUNEL assay of apical spikelet hull cells in WT **(G–I)** and in *lmpa* **(J–L)**. **(M–R)** TUNEL assay of apical spikelet anthers in WT **(M–O)** and in *lmpa* **(P–R)**. TUNEL-positive signals are indicated by the green fluorescence of fluorescein, and nuclei fluoresce deep red signals due to counterstaining with DAPI. le, lemma; pa, palea; C, connective tissue; V, vascular bundle cells; T, tapetum. Bars = 20 μm in **(A–L)**, 50 μm in **(M–R)**.

**Figure 4:**
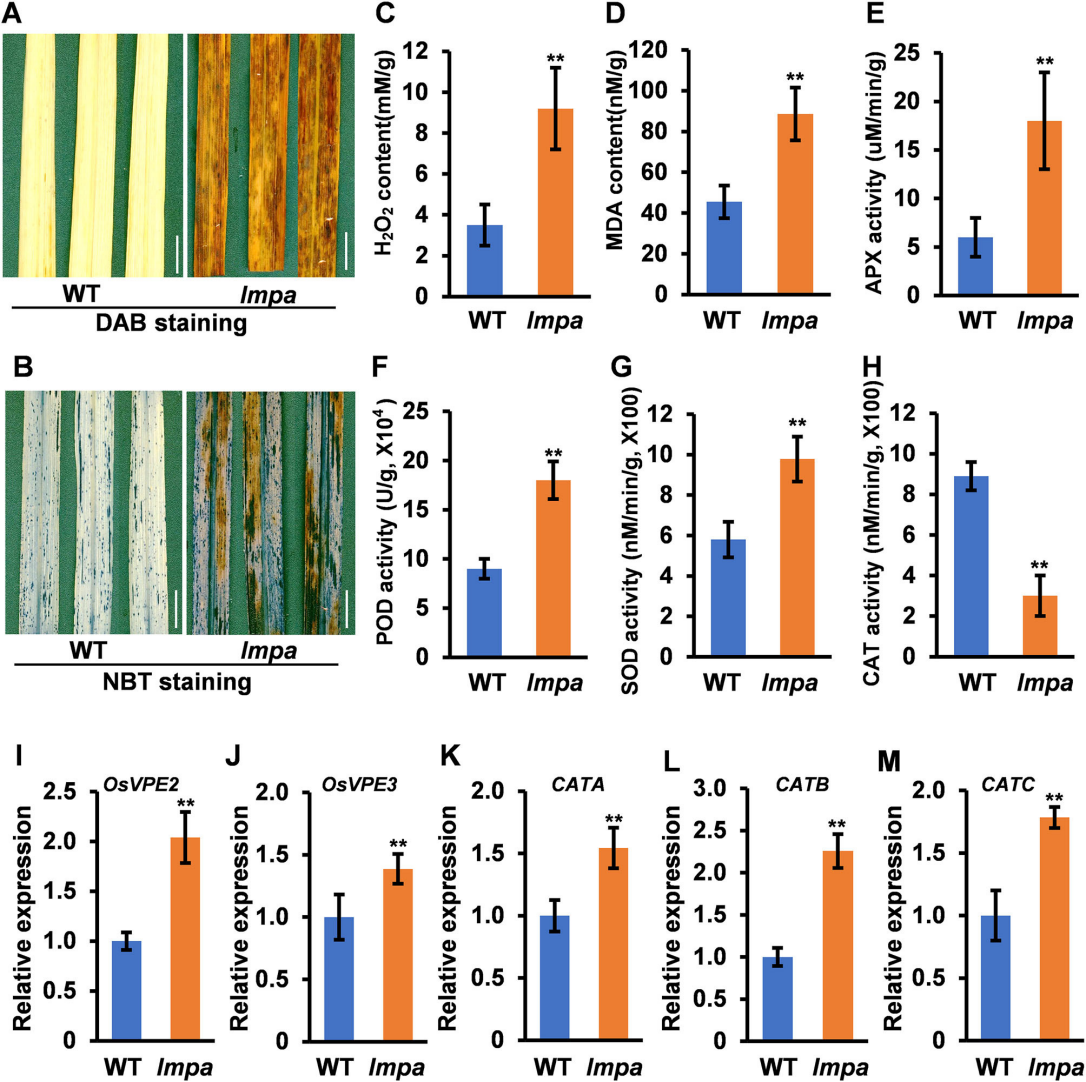
Determination of ROS content in the wild type. **(A)** DAB straining in leaves of WT (left) and *lmpa* (right). **(B)** NBT staining in leaves of WT (left) and *lmpa* (right). **(C–H)** Measurement of H_2_O_2_ content **(C)**, MDA content **(D)**, APX activity **(E)**, POD activity **(F)**, SOD activity **(G)**, and CAT activity **(H)** in leaves. **(I–M)** Expression levels of *OsVPE2* **(I)**, *OsVPE3* **(J)**, *CATA* **(K)**, *CATB* **(L)**, and *CATC* **(M)** in leaves. Error bars represent standard deviation (SD) (*n* = 3); **Significant difference at *p* < 0.01 compared with the WT by Student's *t*-test. Bars = 1 cm in **(A,B)**.

In view of the formation of mimic leaf lesion and degeneration of apical spikelets in *lmpa*, we conducted TUNEL (terminal deoxynucleotidyl transferase-mediated dUTP nick-end labeling) assay to detect cell death. The results showed that TUNEL-positive signals were barely found in WT leaves, while strong signals were observed in *lmpa* (Figures 3A–F). In addition, the strong positive TUNEL signals were also detected in hulls and anthers of apical spikelets in *lmpa*, whereas almost no obvious TUNEL signals were observed in the WT tissues (Figures 3G–R). The excessive accumulation of ROS easily causes oxidative damage, resulting in cell death (Van Breusegem and Dat, 2006). NBT and DAB staining were performed to detect O${}_{2}^{-}$ and H_2_O_2_ accumulation, respectively. The results showed that the in *lmpa*, leaves with lesions were colored deep brown-red by DAB staining and exhibited denser deep-blue spots by NBT staining (Figures 4A,B). In addition, we carried out the measurements of related indicators and found that the levels of H_2_O_2_, MDA, POD, SOD, and APX were significantly higher in *lmpa* than the levels in WT, and the CAT activity was significantly decreased in the *lmpa* mutant (Figures 4C–H). We further examined the expression of PCD representative genes and ROS scavenging-related genes, such as *VPE2, VPE3, CATA, CATB*, and *CATC*, and the results revealed that the expression levels of these genes were significantly increased in *lmpa* (Figures 4I–M).

### *LMPA* Encodes a Plasma Membrane H^+^-ATPases

**Figure 5:**
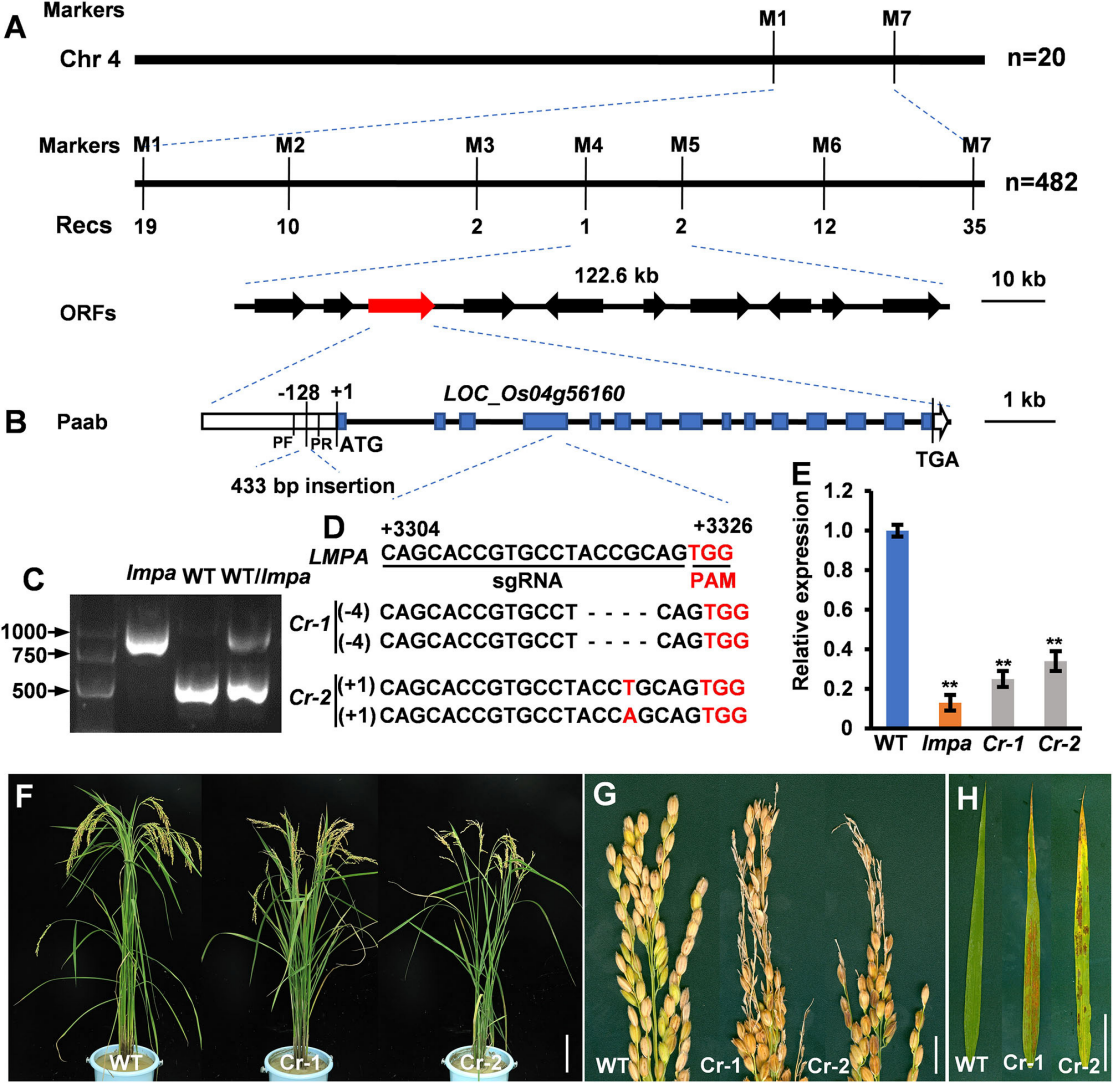
Map-based cloning of *lmpa*. **(A)** Fine mapping of *lmpa*. **(B)** Gene structure of the candidate gene. **(C)** Agarose electrophoresis confirmation of the mutation. DNA fragments were amplified using pF and pR primers indicated in **(B)**. **(D)** Deletion mutation at the target site in two representative knockout lines generated by the CRISPR/Cas9 technology. **(E)** Expression levels of *lmpa* in WT, *lmpa* mutant, and two knockout mutant lines. **(F–H)** Phenotypes of WT and knockout mutant plants. Error bars represent standard deviation (SD) (*n* = 3); **Significant difference at *p* < 0.01 compared with the WT by Student's *t*-test. Bars = 10 cm in **(F)**, 1 cm in **(G,H)**.

To locate the mutant gene, an F_2_ population was constructed by crossing *lmpa* with *indica* cultivar NJ6. With the map-based strategy, the gene was initially mapped on chromosome 4 between the markers M1 and M7 and finally narrowed to a 122.6-kb region between the markers M4 and M5. Ten open reading frames (ORFs) are annotated within the region in the Rice Genome Annotation Project database (http://rice.plantbiology.msu.edu/cgi-bin/gbrowse/rice/) (Figure 5A; Supplementary Table 2). Sequence analysis showed that a 433-bp fragment was inserted into the promoter of *LOC_Os04g56160* in *lmpa* (Figures 5B,C). Compared to the WT, the expression levels of the *LOC_Os04g56160* gene were significantly decreased in *lmpa* (Figure 5E). To verify the gene function, we carried out gene editing by CRISPR/Cas9 technique, and a total of five homozygous knockout lines were obtained. As expected, the expression levels were significantly downregulated in the knockout plants and displayed the lesion mimic phenotype and apical panicle abortion (Figures 5D–H). Based on these findings, we conclude that *LOC_Os04g56160* is *LMPA* gene. According to the Rice Genome Annotation Project, *LMPA* encodes a plasma membrane H^+^-ATPase (PM H^+^-ATPase) and is an allele of *OSA7*.

### The Promoter Activities of *lmpa*

**Figure 6:**
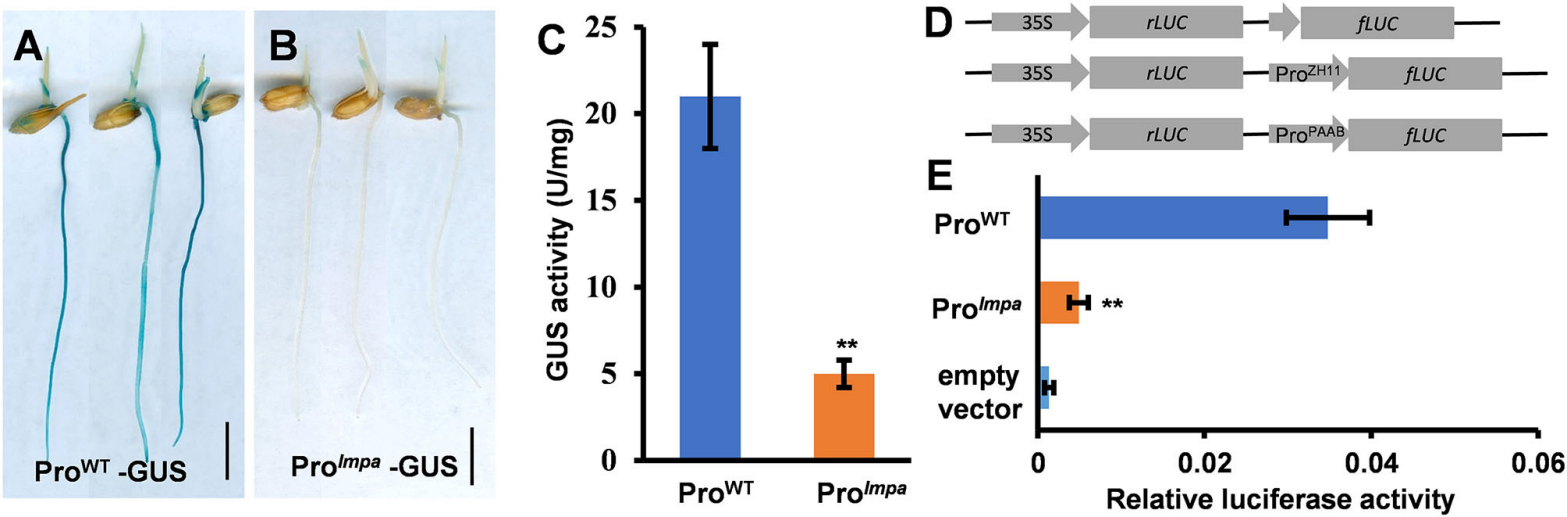
Promoter activity analysis. **(A,B)** GUS staining of Pro^WT^-GUS and Pro^*lmpa*^–GUS transgenic plants. **(C)** The GUS activity in WT and *lmpa* roots at the seedling stage. **(D,E)** Transient expression assays of the WT promoter and *lmpa* promoter. Error bars represent standard deviation (SD) (*n* = 3); **Significant difference at *p* < 0.01 compared with the WT by Student's *t*-test. Bars = 1 cm in **(A,B)**.

To confirm that the fragments inserted in the promoter region affect the expression of *LMPA*, we constructed the *LMPA*::GUS and *lmpa*::GUS reporter genes and introduced them into the WT, respectively. GUS staining indicated that *LMPA* was highly expressed in young roots and coleoptile of the *LMPA*::GUS transgenic lines, while only faint staining was observed in *lmpa*::GUS transgenic plants (Figures 6A,B). We further examined the GUS activity and found that it was significantly higher in *LMPA*::GUS than that of *lmpa*::GUS (Figure 6C). Moreover, the LUC reporter was employed to compare the promoter activity by transient expression in rice protoplasts. The results showed that the WT promoter exhibited higher transcriptional activity than *lmpa* promoter (Figures 6D,E).

### Expression Pattern and Subcellular Localization

**Figure 7:**
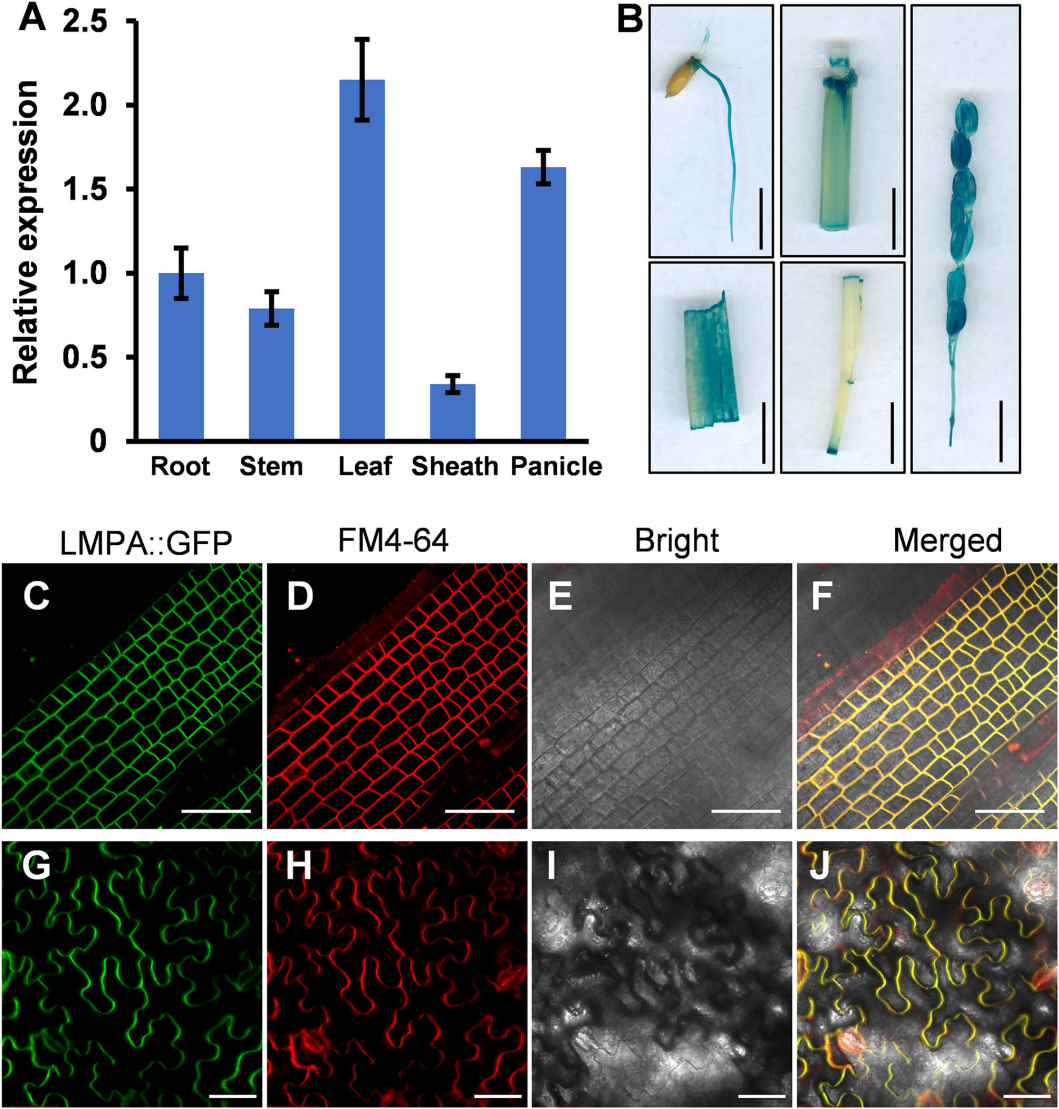
Expression pattern and subcellular localization of the *LMPA* protein. **(A)** Relative expression of *LMPA* in various tissues. **(B)** Tissue-specific expression of the GUS gene driven by the *LMPA* promoter. **(C–J)** Plasma membrane localization of *LMPA* in *LMPA-GFP* transgenic plants **(C–F)** and leaf cells of *N. benthamiana* **(G–J)**. FM4-64 was used as a membrane marker. Error bars represent standard deviation (SD) (*n* = 3). Bars = 1 cm in **(B)**, 50 μm in **(C–J)**.

To investigate the spatial expression pattern of *LMPA*, the qRT-PCR was employed to measure the expression in various organs, including root, stem, leaf, leaf sheath, and panicle. The results indicated that *LMPA* was expressed in all the investigated tissues, particularly higher in leaves and panicles (Figure 7A). Moreover, the GUS reporter gene driven by the native promoter was expressed in the WT background. Strong GUS staining was mainly detected in roots, stems, leaves, and panicles, which was consistent with the results obtained by qRT-PCR analysis (Figure 7B).

To determine the subcellular localization of *LMPA* protein, we constructed an LMPA-GFP fusion protein driven by the *Ubiquitin1* promoter and expressed protein in rice protoplasts. The GFP fluorescence was observed at plasma membranes and colocalized with the FM4-64 marker (Figures 7C–F). In addition, the *pubi*::LMPA-GFP plasmid was introduced into *N. benthamiana* leaves. The result indicated that the GFP fluorescence was also localized in the plasma membranes (Figures 7G–J).

### Low Temperature Rescues the Lesion Mimic Phenotype of *lmpa*

**Figure 8:**
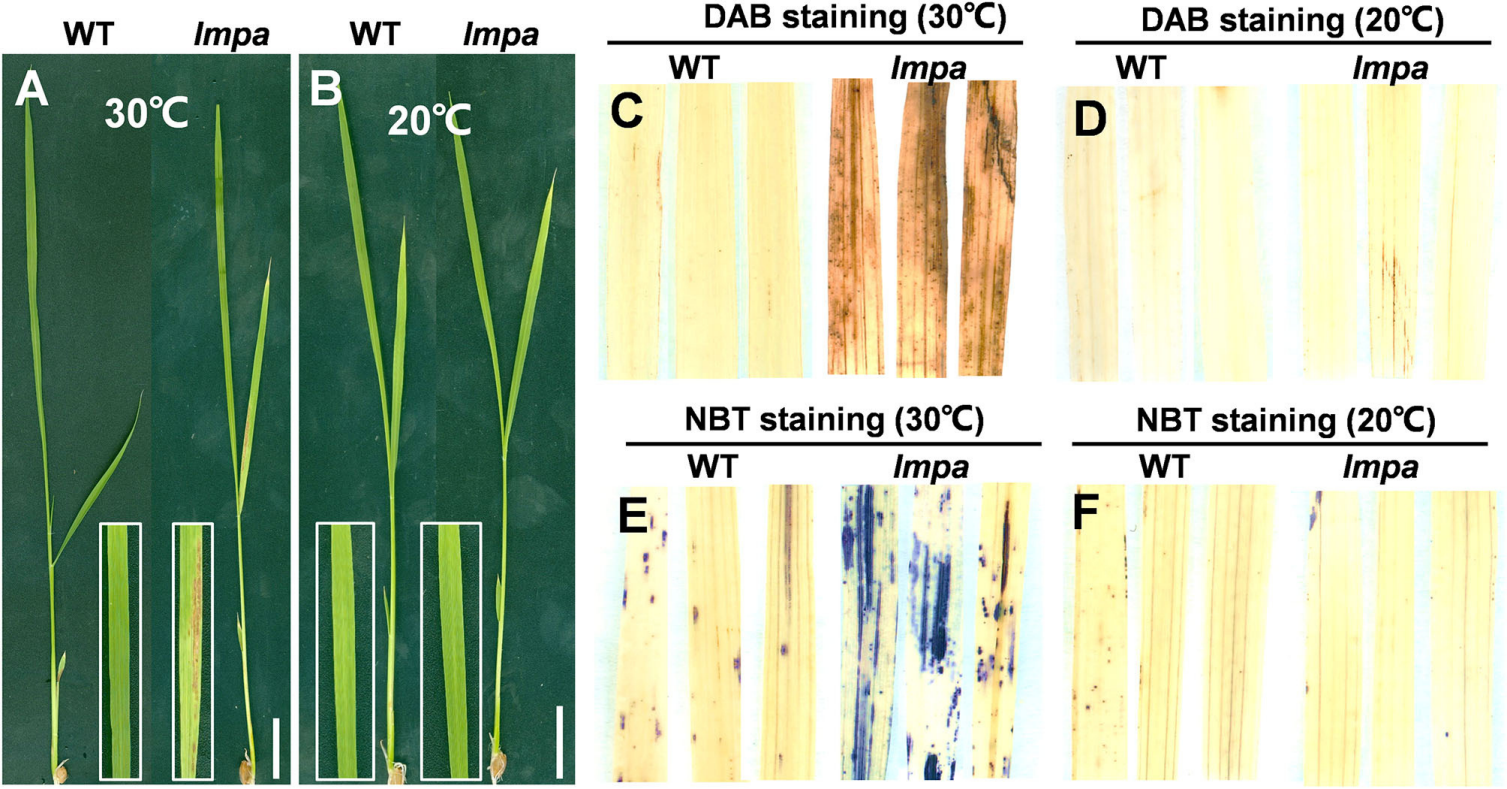
Temperature treatment, and DAB and NBT staining. **(A,B)** Phenotypes of WT and *lmpa* mutant under different temperatures. **(C,D)** DAB staining of leaves grown at 30°C **(C)** and 20°C **(D)**. **(E,F)** NBT staining of leaves grown at 30°C **(E)** and 20°C **(F)**. Bars = 2 cm in **(A,B)**.

Extreme environmental conditions, such as high temperature, high humidity, strong light, and low nitrogen levels, often induce lesion mimic symptoms and lead to the panicle apical abortion during the rice growth (Yao et al., 2000; Zhang et al., 2017; Cui et al., 2021; Xia et al., 2022). We performed the temperature treatments and found that the *lmpa* mutant was sensitive to high temperatures. Under the 30°C conditions, the reddish-brown lesions were scattered on the surface of leaves and spread gradually with growth in the *lmpa* mutants. However, there were no obvious lesions on the leaf surface in *lmpa* when grown under low temperatures (20°C) (Figures 8A,B). Meanwhile, the NBT and DAB staining techniques were applied to detect the excessive accumulation of ROS. Under normal conditions, the *lmpa* leaves were stained with deep brown-red and deep blue spots by DAB and NBT staining, respectively, whereas the staining was minimal at low temperatures (Figures 8C–F). In addition, we detected the expression levels of LMPA when the plants were grown at different temperatures. The results showed that the expression levels of *LMPA* were significantly reduced in *lmpa* mutant compared to WT when grown at both 20 and 30°C, but the expression level in *lmpa* mutants grown at 20°C was higher than that observed at 30°C (Supplementary Figure 5). These results indicated that the lesion mimic phenotype of *lmpa* can be rescued by low temperature.

### Disruption of *LPMA* Impairs the Salt Stress Tolerance

Plasma membrane H^+^-ATPases are the primary active transporters that pump protons out of the cell and mediate Na^+^ extrusion by providing proton motive force (Vitart et al., 2001). Therefore, PMAs play an important role in salt tolerance in a plant. In this study, we performed salt treatments and found that the disruption of *LPMA* impairs the salt stress tolerance in rice. When 7-day-old WT and *lmpa* seedlings were treated with different concentrations of NaCl (75 mM and 150 mM) for 7 days and recovered after 7 days, only a few WT leaves showed a wilted phenotype, while the majority of *lmpa* leaves exhibited a markedly wilted phenotype, especially under high concentrations of NaCl (Supplementary Figures 6A–F). In addition, the survival rate of WT was about 78.9% and 41.3% but that of the mutants was only 47.1% and 14.4%, respectively, after 7 days of recovery (Supplementary Figure 5G). These findings suggest that *LPMA* is important for salt stress tolerance in rice.

## Discussion

In this study, we isolated and characterized a rice mutant *lmpa*, which displayed lesion mimic leaf and the apical spikelet degeneration phenotype. The panicle apical abortion is observed when the panicle length reaches 5 cm during panicle development, indicating that *LMPA* is required for maintaining the panicle development (Figure 1D; Supplementary Figure 2). To date, several genes responsible for panicle apical degeneration in rice have been reported, such as *TUT1/ES1, OsCIPK31, SPL6, OsALMT7, DPS1, OsASA*, and *PAA3* (Bai et al., 2015; Heng et al., 2018; Peng et al., 2018; Wang Q. L. et al., 2018; Yang et al., 2021; Zhou et al., 2021). With the exception of the panicle abortion, other phenotypes, such as pollen sterility, low seed setting rate, smaller grain size, and early leaf senescence, were also observed in these mutants. Similar to these mutants, the *lmpa* leaf showed lesion mimic phenotype during the whole life cycle (Figures 4, 8). Therefore, these results suggest that the genes related to panicle degradation not only regulate panicle development but also exhibit extensive effects on the growth of rice plants.

The PMA is a transmembrane glycoprotein that mediates ATP-dependent H^+^ extrusion from the cytosol into the extracellular space and activates the secondary transporters or ion channels (Toda et al., 2016; Hoffmann et al., 2020; Loss Sperandio et al., 2020; Lee et al., 2021; Zhang et al., 2021). In this study, we show that *LMPA* encodes a plasma membrane H^+^-ATPase, which is an allele of *OsA7*. The mutant *osa7* was obtained by *Tos17* insertion and exhibited a phenotype showing severe growth defects (Toda et al., 2016). Unlike *osa7* and *paa-h* mutants (Akter et al., 2014), the *lmpa* mutant is derived from insertion mutations in the promoter, which leads to a decrease in promoter activity. With the exception of the panicle abortion, the *lmpa* leaves synchronously showed lesion mimic phenotype (Figures 5F,G). In the present study, the *lmpa* phenotypes may be caused by the different mutations of the *LMPA*/*OsA7* gene and different genetic backgrounds. Thus, *LMPA* may play a role in many aspects of plant growth and development.

Nutrient deficiency or adverse environmental conditions often induce panicle abortion and leaf lesion mimic symptoms (Huang et al., 2011; Tan et al., 2011; Kobayasi et al., 2015; Zhang et al., 2017; Wang Z. et al., 2018; Liu et al., 2019). In this study, we found that *lmpa* is sensitive to high temperature and that the leaf lesion mimic phenotypes can be rescued by low-temperature treatment. In addition, the expression level of *LMPA* was higher in *lmpa* mutants grown at 20°C than those grown at 30°C (Supplementary Figure 5). Therefore, we believe that *LMPA* expression is inhibited by high temperature in the *lmpa* mutant, and low temperature could partially relieve this inhibition. This may explain why the mutant phenotype was rescued under low temperature. Moreover, *lmpa* is more sensitive to high salt stress (Supplementary Figure 6). PMAs are activated under salt stress and provide the driving force for Na^+^/H^+^ antiporter (Haruta and Sussman, 2012; Yang et al., 2019). Taken together, we speculate that *lmpa* mutation may fail to maintain the intracellular and extracellular proton gradients, which subsequently affects the transmembrane transport.

Reactive oxygen species are known to be an important trigger factor of PCD, and the excessive accumulation of ROS makes cell membranes highly oxidized, which further affects cell permeability and ultimately leads to cell death (Mittler, 2017; Mhamdi and Van Breusegem, 2018; Waszczak et al., 2018). *OsALMT7* encoded an aluminum-activated malate transporter, and the malate could maintain the balance of intracellular redox potential by participating in the redox reactions to produce NAD(H) or NADP(H). The loss of function of *OsALMT7* mutant leads to the malate reduction, which might disrupt the redox balance in panicle cells, thus leading to ROS accumulation and induced cell death (Heng et al., 2018). NADPH oxidase-plasma membrane H^+^-ATPase positive feed-forward is involved in ROS-mediated chloroplast avoidance (Majumdar and Kar, 2020). In this study, the TUNEL assay showed that the PCD occurred in degenerated spikelets and lesion mimic leaves (Figure 3). Histochemical staining and determination of ROS-related indicators showed that ROS excessively accumulated under normal conditions in *lmpa*, but there was no significant difference when compared to WT under low temperatures (Figures 4, 8). In addition, the expression levels of the representative genes of PCD and genes related to ROS scavenging were significantly increased (Figure 4). These results indicated that the induction of PCD due to excessive accumulation of ROS in panicles and leaves might be the reason for the panicle degeneration and lesion mimic symptoms. Taking these findings together, we speculate that the mutation in *LMPA* causes the imbalance of ROS signals in chloroplast and that high temperature alters the expression of genes related to ROS scavenging, further reducing the ability to scavenge ROS and resulting in the excessive accumulation of ROS and ultimately inducing cell death. However, the molecular mechanism of *LMPA* in this process needs to be further studied in the future, which can also help us to further understand the function of H^+^-ATPase in the plasma membrane.

## Data Availability Statement

The original contributions presented in the study are included in the article/Supplementary Material, further inquiries can be directed to the corresponding authors.

## Author Contributions

JH and QQ designed and supervised research studies. PH and YWe wrote the manuscript. PH, YWe, and YT performed experiments. PH, YT, YWa, HW, JW, KW, and BC contributed to the phenotype and data analysis. LS, YF, LZ, DR, ZG, GZ, DZ, and DX provided technical assistance. All authors have read and agreed to the published version of the manuscript.

## Funding

This study was supported by the Zhejiang Province Outstanding Youth Fund (LR19C130001), Natural Science Foundation of China (31871594), National Science Foundation of Zhejiang Province (LY19C130001), and the Open Foundation of State Key Laboratory of Rice Biology (20190103).

## Conflict of Interest

The authors declare that the research was conducted in the absence of any commercial or financial relationships that could be construed as a potential conflict of interest. The reviewer YX declared a shared affiliation with the author(s) YWe to the handling editor at the time of review.

## Publisher's Note

All claims expressed in this article are solely those of the authors and do not necessarily represent those of their affiliated organizations, or those of the publisher, the editors and the reviewers. Any product that may be evaluated in this article, or claim that may be made by its manufacturer, is not guaranteed or endorsed by the publisher.

## Supplementary Material

Supplementary Figure 1 Leaf phenotype of WT and *lmpa* mutant at seeding stage. **(A,B)** leaf phenotype in WT. **(C,D)** leaf phenotype in *lmpa* mutant. Bars =10 cm in **(A,C)**, 2 cm in **(B,D)**.

Supplementary Figure 2 Characteristics of spikelets in WT and *lmpa*. **(A–F)** Representative images of WT (left) and *lmpa* (right) developing panicles at different stage, 1 cm **(A)**, 3 cm **(B)**, 5 cm **(C)**, 7 cm **(D)**, 10 cm **(E)**, 15 cm **(F)**. **(A1–F1)** Representative WT spikelets from the top of the corresponding panicles shown in **(A–F)**. **(A2–F2)** Representative *lmpa* spikelets from the top of the corresponding panicles shown in **(A–F)**. Bars =1 cm in **(A–F)**, 2 mm in **(A1–F1,A2–F2)**.

Supplementary Figure 3 Spikelet morphology of WT and *lmpa* mutant. **(A)** Morphology of WT and *lmpa* mutant panicle at anthesis stage. **(B)** Spikelet morphology of apical and middle spikelets in WT and *lmpa* mutant. **(C)** Anther and pistil phenotype of apical and middle spikelets in WT and *lmpa* mutant. **(D)** Potassium iodide staining of mature pollen grains of apical and middle spikelets in WT and *lmpa* mutant. **(E)** Grain size of apical and middle spikelets in WT and *lmpa* mutant. Bars = 2 cm in **(A)**, 2 mm in **(B,C,E)**, and 100 μm in **(D)**.

Supplementary Figure 4 Ultrastructural analysis of chloroplast and determination of chlorophyll (Chl) content at heading stage. **(A–D)** TEM was used to detect the chloroplasts of WT and *lmpa* leaves, WT leaves **(A,B)**; *lmpa* leaves **(C,D)**. **(E)** The Chl content in WT and *lmpa* leaves at heading stage. C, chloroplast; G, granum; OG, osmiophilic granule. Error bars represent standard deviation (SD) (*n* = 3); ^**^Significant difference at *p* < 0.01 compared with the WT by Student's *t*-test. Bars = 10 cm in **(A)**, 5 cm in **(B)**, 2 cm in **(C)**, and 1 cm in **(D)**. Bars = 1 μm in **(A,C)**, 0.5 μm in **(B,D)**.

Supplementary Figure 5 Relative expression levels of *LMPA* in the WT and *lmpa* grown at 30 and 20°C, respectively. Error bars represent standard deviation (SD) (*n* = 3); ^**^Significant difference at *p* < 0.01 compared with the WT by Student's *t*-test.

Supplementary Figure 6 Disruption of *LMPA* decreases salt tolerance in rice seedlings. **(A–C)** Phenotype of WT and *lmpa* mutant treated by salt stress for 7 days at seeding stage, CK **(A)**, 75 mM **(B)**, 150 mM **(C)**. **(D–F)** Phenotype of WT and *lmpa* mutant after recovery for 7 days. **(G)** Survival rate of rice seedlings after recovery for 7 days. Error bars represent standard deviation (SD) (*n* = 3); ^**^Significant difference at *p* < 0.01 compared with the WT by Student's *t*-test.

Supplementary Table 1 Primers used in this study.

Supplementary Table 2 List of genes in the 122.6-kb target region.
