# Supplementary material for: LMPA Regulates Lesion Mimic Leaf and Panicle Development Through ROS-Induced PCD in Rice
Source: Front Plant Sci. 2022 May 2;13:875038. doi: 10.3389/fpls.2022.875038 (PMC9108926; doi:10.3389/fpls.2022.875038)

**Supplemental Figure 1.** Leaf phenotype of WT and *lmpa* mutant at seeding stage.

(A, B) leaf phenotype in WT. (C, D) leaf phenotype in *lmpa* mutant*.* Bars=10 cm in (A,C), 2 cm in (B, D).


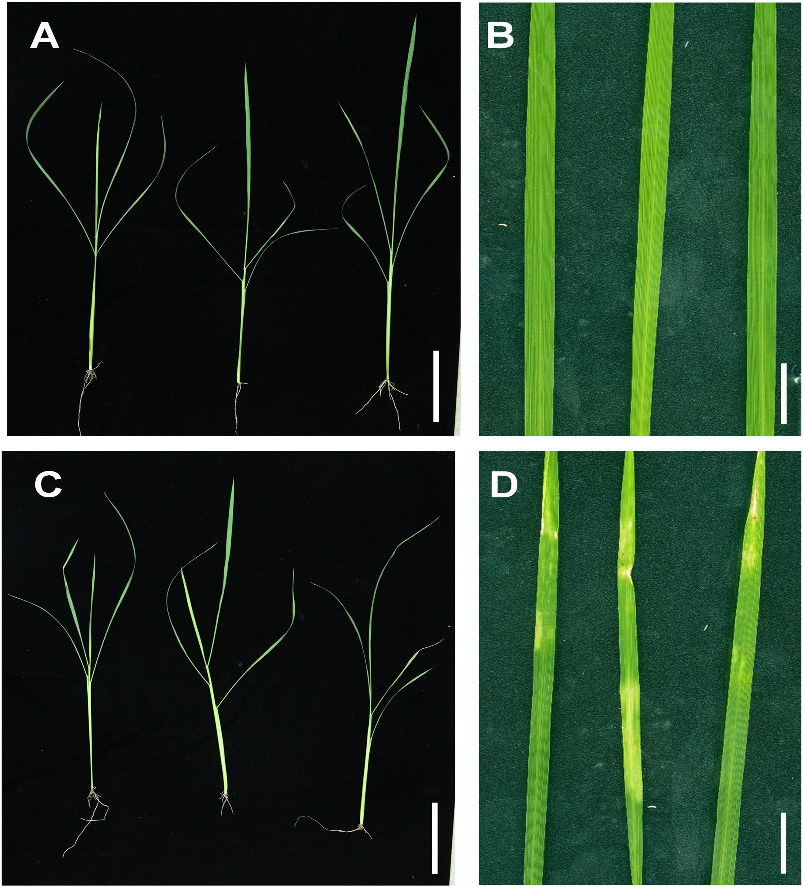


**Supplemental Figure 2.** Characteristics of spikelets in WT and *lmpa.*

(A-F) Representative images of WT (left) and *lmpa* (right) developing panicles at different stage, 1cm (A), 3 cm (B), 5 cm (C), 7 cm (D), 10 cm (E), 15 cm(F). (A1-F1) Representative WT spikelets from the top of the corresponding panicles shown in (A) to (F). (A2-F2) Representative *lmpa* spikelets from the top of the corresponding panicles shown in (A) to (F). Bars=1 cm in (A-F), 2 mm in (A1-F1, A2-F2).


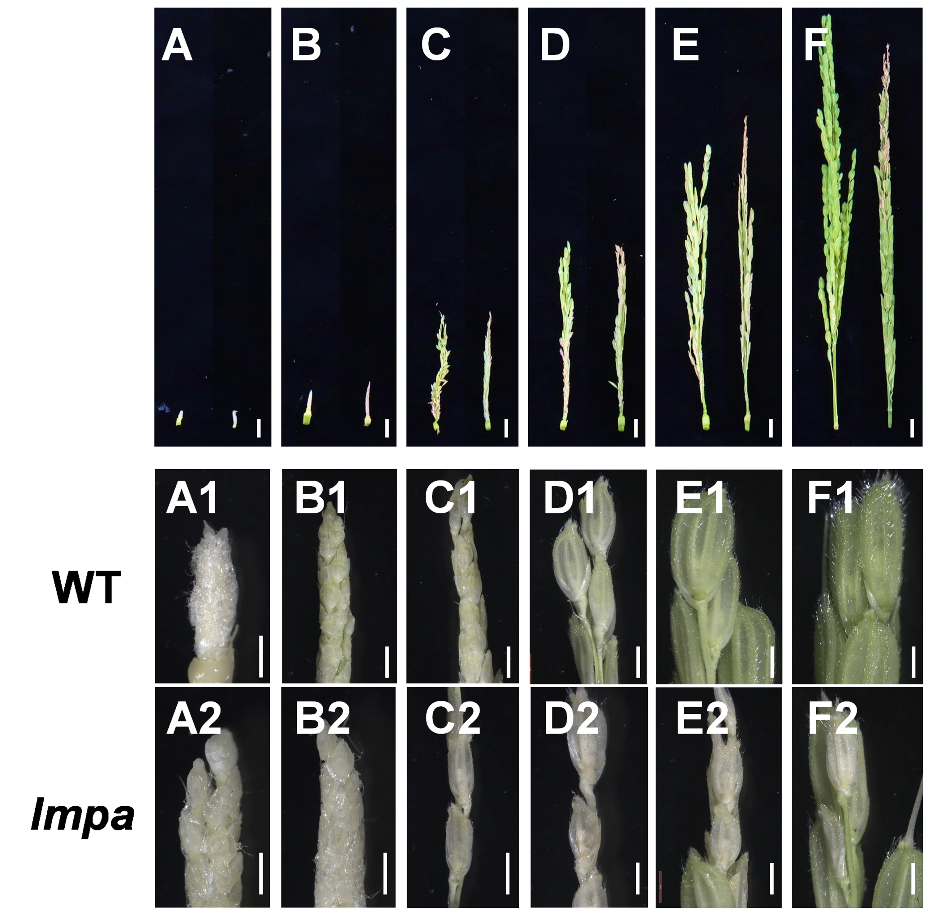


**Supplemental Figure 3.** Spikelet morphology of WT and *lmpa* mutant.

(A) Morphology of WT and *lmpa* mutant panicle at anthesis stage. (B) Spikelet morphology of apical and middle spikelets in WT and *lmpa* mutant. (C) Anther and pistil phenotype of apical and middle spikelets in WT and *lmpa* mutant. (D) Potassium iodide staining of mature pollen grains of apical and middle spikelets in WT and *lmpa* mutant. (E) Grain size of apical and middle spikelets in WT and *lmpa* mutant. Bars=2 cm in (A), 2 mm in (B,C,E), and 100 μm in (D).


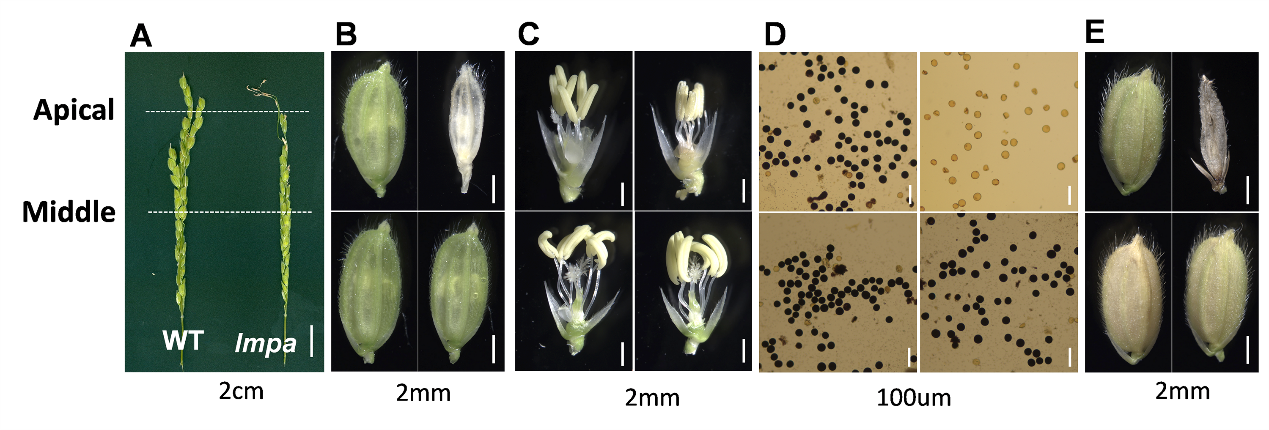


**Supplemental Figure 4.** Ultrastructural analysis of chloroplast and determination of chlorophyll (Chl) content at heading stage*.*

(A-D) TEM was used to detect the chloroplasts of WT and *lmpa* leaves, WT leaves (A, B); *lmpa* leaves (C, D). (E) The Chl content in WT and *lmpa* leaves at heading stage. C, chloroplast; G, granum; OG, osmiophilic granule. Error bars represent standard deviation (SD) (n=3); ** Significant difference at p < 0.01 compared with the WT by Student’s *t*-test. Bars=10 cm in (A), 5 cm in (B), 2 cm in (C), and 1 cm in (D). Bars=1 μm in (A, C), 0.5 μm in (B, D).


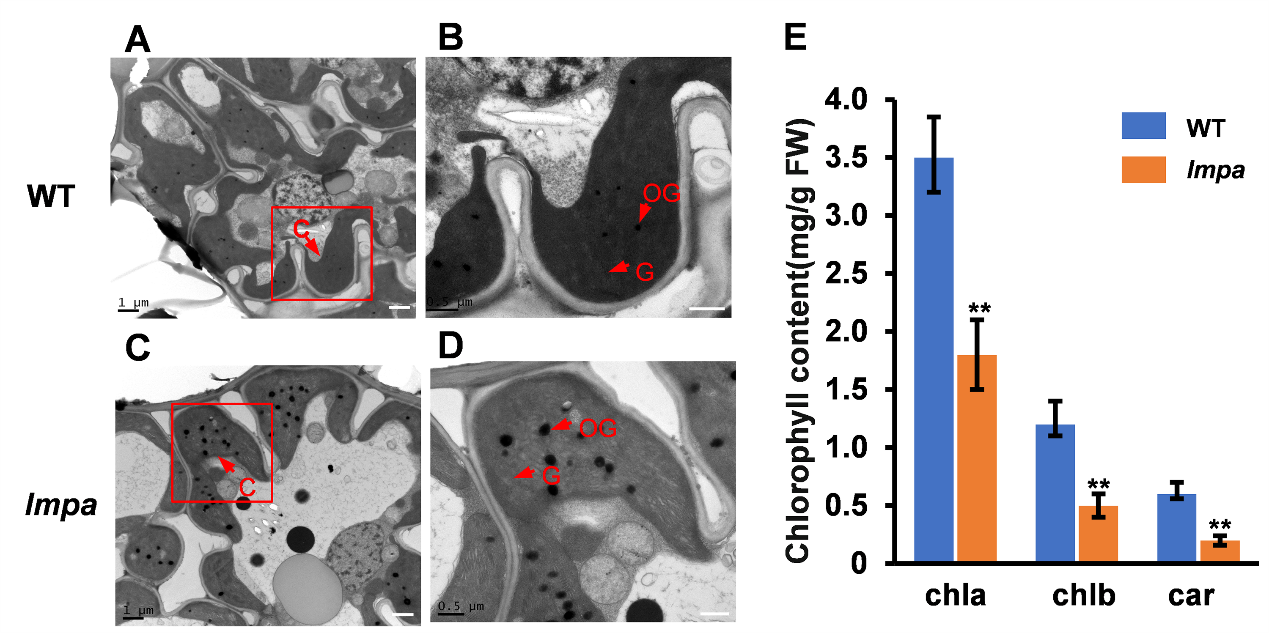


**Supplemental Figure 5.** Relative expression levels of *LMPA* in the WT and *lmpa* grown at 30°C and 20°C, respectively. Error bars represent standard deviation (SD) (n=3); ** Significant difference at p < 0.01 compared with the WT by Student’s *t*-test.


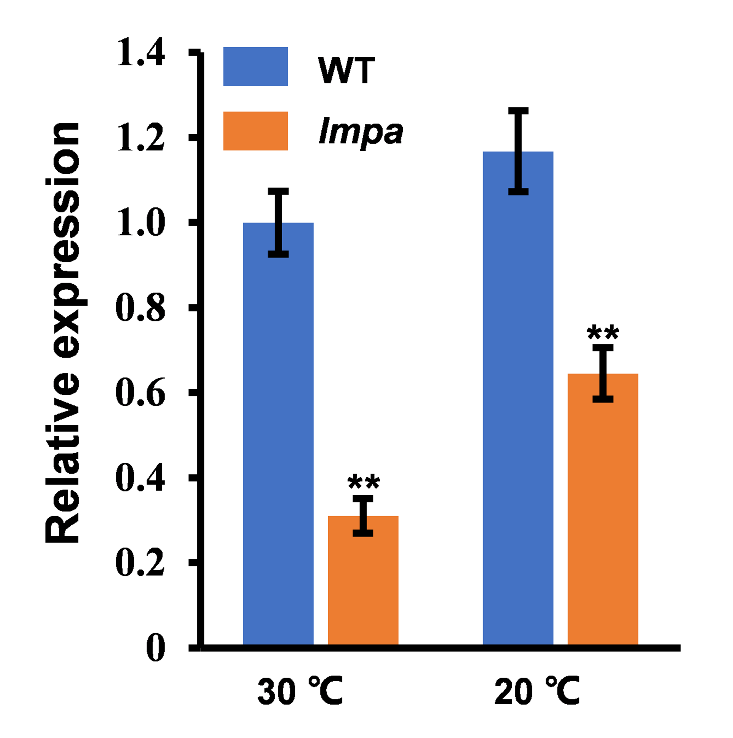


**Supplemental Figure 6.** Disruption of *LMPA* decreases salt tolerance in rice seedlings.

(A-C) Phenotype of WT and *lmpa* mutant treated by salt stress for 7 days at seeding stage, CK(A), 75 mM (B), 150 mM (C). (D-F) Phenotype of WT and *lmpa* mutant after recovery for 7 days. (G) Survival rate of rice seedlings after recovery for 7 days. Error bars represent standard deviation (SD) (n=3); ** Significant difference at p < 0.01 compared with the WT by Student’s *t*-test.


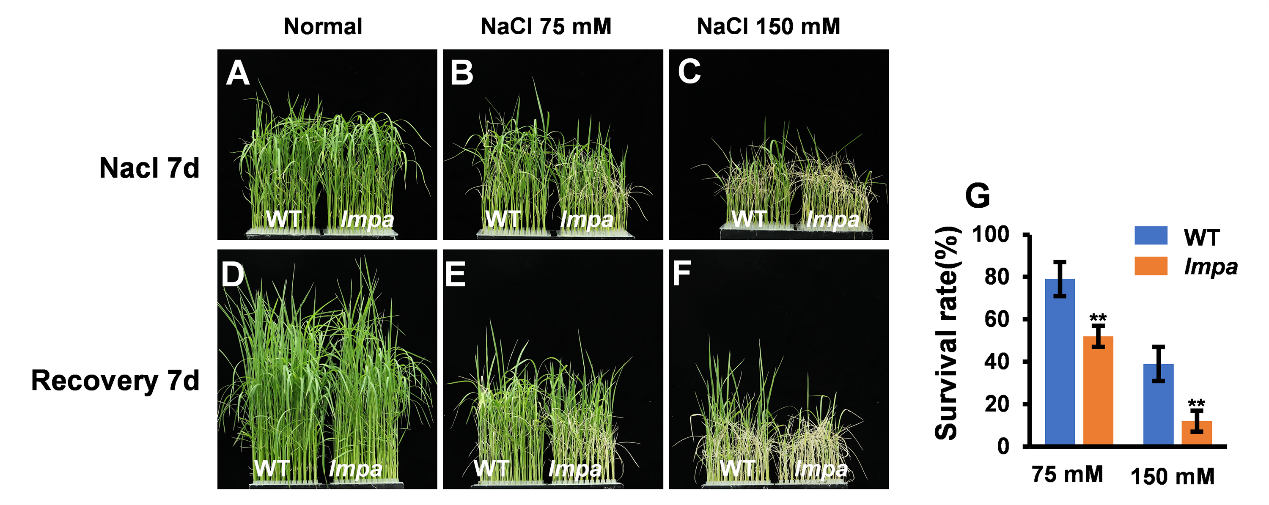

Supplement: Supplementary Figure 1 — Leaf phenotype of WT and lmpa mutant at seeding stage. (A,B) leaf phenotype in WT. (C,D) leaf phenotype in lmpa mutant. Bars =10 cm in (A,C), 2 cm in (B,D). [file Data_Sheet_1.docx]
